# Comparative analysis of lincRNA in insect species

**DOI:** 10.1186/s12862-017-0985-0

**Published:** 2017-07-03

**Authors:** Alberto Lopez-Ezquerra, Mark C. Harrison, Erich Bornberg-Bauer

**Affiliations:** Institute of Evolution and Biodiversity, University of Münster, Hüfferstrasse,1, Münster, Münster, Germany

**Keywords:** lincRNA, RNA secondary structure, Evolution, Transcriptomics

## Abstract

**Background:**

The ever increasing availability of genomes makes it possible to investigate and compare not only the genomic complements of genes and proteins, but also of RNAs. One class of RNAs, the long noncoding RNAs (lncRNAs) and, in particular, their subclass of long intergenic noncoding RNAs (lincRNAs) have recently gained much attention because of their roles in regulation of important biological processes such as immune response or cell differentiation and as possible evolutionary precursors for protein coding genes. lincRNAs seem to be poorly conserved at the sequence level but at least some lincRNAs have conserved structural elements and syntenic genomic positions. Previous studies showed that transposable elements are a main contribution to the evolution of lincRNAs in mammals. In contrast, plant lincRNA emergence and evolution has been linked with local duplication events. However, little is known about their evolutionary dynamics in general and in insect genomes in particular.

**Results:**

Here we compared lincRNAs between seven insect genomes and investigated possible evolutionary changes and functional roles. We find very low sequence conservation between different species and that similarities within a species are mostly due to their association with transposable elements (TE) and simple repeats. Furthermore, we find that TEs are less frequent in lincRNA exons than in their introns, indicating that TEs may have been removed by selection. When we analysed the predicted thermodynamic stabilities of lincRNAs we found that they are more stable than their randomized controls which might indicate some selection pressure to maintain certain structural elements. We list several of the most stable lincRNAs which could serve as prime candidates for future functional studies. We also discuss the possibility of de novo protein coding genes emerging from lincRNAs. This is because lincRNAs with high GC content and potentially with longer open reading frames (ORF) are candidate loci where de novo gene emergence might occur.

**Conclusion:**

The processes responsible for the emergence and diversification of lincRNAs in insects remain unclear. Both duplication and transposable elements may be important for the creation of new lincRNAs in insects.

**Electronic supplementary material:**

The online version of this article (doi:10.1186/s12862-017-0985-0) contains supplementary material, which is available to authorized users.

## Background

Widespread transcription beyond protein coding regions produces several types of noncoding RNAs [1] such as long noncoding RNAs (lncRNAs). lncRNAs are a type of noncoding RNA that can be defined as transcripts longer than 200 nucleotides but which lack canonical open reading frames (ORFs) [2]. lncRNAs may overlap in antisense orientation of genes or lie within introns or intergenic regions. lncRNAs are transcribed by RNA polymerase II and tend to be polyadenylated and spliced. lncRNAs serve a plethora of biological functions in different cellular locations, although most lncRNAs act in the nucleus or in the cytoplasm [3]. Most lncRNAs show low sequence conservation and thus they can not be predicted reliably from genomic sequences [4]. Therefore, RNA-seq data are generally used to detect lncRNA transcription. The majority of lncRNAs are not functionally characterised, but some lncRNAs are well described, mainly in model organisms. One example is Rox RNA in *Drosophila melanogaster*, required for dosage compensation [5] or lncRNA HOTAIR described in humans as a cancer-related lncRNA containing a modular secondary structure [6]. lncRNAs that are located between two protein coding genes are termed long intervening RNAs (lincRNAs) [1]. lincRNAs, the main focus of this work, have focused strong research interest due to their detection in RNA-seq studies and ease of study in comparison to lncRNA that overlap coding genes [1].

There are several possible scenarios for explaining the origin of lincRNAs. lincRNAs might have emerged from protein coding genes that became “pseudogenized” [7]. Alternatively, emergence through duplication from another gene or lincRNA is possible, although unlikely in vertebrates as recently investigated [4]. In contrast, in plants whole genome duplication appears to be an important mechanism for the diversification of lincRNA repertoires. De novo evolution of new lincRNAs from intergenic regions might occur [8]. Intergenic regions might acquire elements such as transposable elements that allow independent transcription. The contribution of transposable elements (TE) to the evolution and functionality of lincRNAs has been examined in several studies [4, 9–11]. Transposable elements might give functional domains [10] to lncRNAs, providing transcription start sites (TSS), splice sites and poly-A sites [11]. Some evidence indicates that TEs tend to be more frequent in young lincRNAs [12] indicating that TEs might be important for the emergence of lincRNAs. TEs might also provide lincRNAs with protein binding sites, DNA or RNA binding sites as well as residues essential for the formation of secondary structures [13, 14].

Recent studies have used RNA-seq data to assemble lincRNAs since prediction of lincRNAs from genomic sequences alone is generally not reliable without experimental evidence of transcription [4, 15, 16]. Therefore, to compare lincRNAs in different species, lincRNAs have to be assembled separately for each species and common patterns analyzed afterwards [4]. Consequently, most lincRNAs which are available in databases have been detected in RNA-seq studies. Due to the lower expression level of lincRNAs in comparison to coding genes, lncRNAs require a higher read depth for detection [17]. The relatively low agreement of the lincRNAs between different studies might be related to the different tissues and conditions from which the data were obtained or different computational strategies for their identication but might also be an indication of erroneous lncRNA annotations. For example 11,810 lncRNAs (6250 lincRNAs) were identified in the lepidopteran *Bombyx mori* [18] whereas the number in other insect species is less than half the amount: *Plutella xylostella* (3844 lincRNAs), *Anopheles gambiae* (2059 lincRNAs), *Aedes aegypti* (3482 lincRNAs), *Apis mellifera* (1514 lincRNAs). Different computational strategies and arbitrary filtering criteria for the identification of a true lincRNA in contrast to just transcriptional noise lead to lincRNA annotations that are not easily comparable. This is especially true for monoexonic transcripts which might in some cases just be mapping artifacts. For this reason some studies exclude the analysis of monoexonic lincRNAs altogether, considering them in most cases to be mapping artifacts [4, 19, 20].

While many studies on the properties and evolution of lincRNAs have focused on mammalian species (recently reviewed in [21]), some studies have examined lincRNAs in different insect genomes. Some focused only on lincRNAs while others also analyzed antisense and intronic lncRNAs. The first study that established a methodology for the identification of lincRNAs from RNA-seq data in insects was by Young et. al in 2012 [16]. This study led to the discovery of more than a thousand lincRNAs in the *Drosophila melanogaster* genome. Subsequently several further groups have published lincRNAs from *Anopheles gambiae* [22], *Aedes aegypti* [23], *Apis mellifera* and *Apis cerana*, [24] *Drosophila pseudoobscura*, [25] the lepidopterans *Plutella xylostella* [26] and *Bombyx mori* [18].

To the best of our knowledge no detailed comparative study on the properties of lincRNAs in insect species is available. Thus in this study publicly available datasets of lincRNAs and custom assembled lincRNAs from seven insect species are compared and different properties are analysed to better understand the biological roles of lincRNAs. Insects are very useful models for genome research since they possess a small genome in comparison with mammalian species thus speeding up genomic analyses, and allowing insights that can be extrapolated to other species with bigger and more complex genomes [27]. A pipeline for the detection of lincRNAs from RNA-seq data comparable to other recent studies [4] was implemented in this study. Subsequently, lincRNA properties in terms of structure, sequence composition, conservation, overlap with repetitive elements were analysed. By comparative analysis of lincRNAs in different insect species we expected to gain new insights into the properties, evolution and potential roles of lincRNAs.

## Methods

### lincRNA sequences analysed

In order to study the properties of lincRNAs in different insect species, species with a high quality genome and ideally with RNA-seq data from different tissues available in public databases were required [28]. lincRNAs from two different species were assembled (*Tribolium castaneum* and *Nasonia vitripennis*) and publicly available assemblies of lincRNAs for *Drosophila melanogaster*, *Drosophila pseudoobscura*, *Anopheles gambiae*, *Apis cerana* and *Apis mellifera* were used.

Publicly available RNA-seq data were used to assemble a set of lincRNAs from *N. vitripennis* and *T. castaneum*. RNA-seq data from different tissues were obtained from the Sequence Read Archive (SRA) [28]. RNA-seq reads were processed by Trimmomatic [29] to remove low-quality reads and mapped to the genomes of *Tribolium castaneum* (Tcas 3.0) and *Nasonia vitripennis* (v1.2). Reads were mapped to the genomes using the splice aware aligner Tophat2 [30], transcripts were constructed for each tissue sample using Cufflinks [31] and all the gff files were merged using the Cuffmerge [31] tool. Transcripts that did not overlap with annotated protein coding genes were removed by selecting Cufflinks transcripts of class code “i”. These correspond to intergenic transcripts. Since some of these might have been pseudogene remnants we filtered out those with high coding potential using the CPC [32] tool. Furthermore, to remove pseudogenes remnants, lincRNAs that contained matches to protein domains from the Pfam-A [33] database or Blastx hits with an e-value lower than 10^-6^ were also filtered out. Previously assembled lincRNAs from *Drosophila melanogaster* [34], *Apis mellifera* [24], *Apis cerana* [24], *Drosophila pseudoobscura* [25] and *Anopheles gambiae* [17] were downloaded and gff coordinates processed to obtain fasta sequences using the gffread utility function from Cufflinks [31].

### RNA structure analysis

RNAfold from the Vienna RNA package [35] was used to calculate the minimum free energy (MFE) of each sequence. Shuffled RNA sequences maintaining dinucleotide composition were used as background control. Shuffled RNA sequences were obtained by using the dinucleotide shuffle algorithm of Altschul-Erikson [36] as implemented by P.Grote in the MEME suite [37]. The ratio of MFE of the native lincRNAs compared to the average of 100 dinucleotide controls was calculated.

The folding strength of the lincRNAs was calculated as proposed in [38]. Folding strength represents the fraction of nucleotides that are paired in an RNA molecule. The folding strength provides information of the likelihood of each nucleotide being paired in the ensemble of secondary structures. Z-scores of folding strength for each lincRNA were obtained using the ration $Z=\frac {x - \mu }{\sigma }$ with x representing the value of folding strength for each lincRNA, *μ* the average folding strength of 100 shuffled controls and *σ* the standard deviation of the values obtained for the shuffled controls.

Additionally, ParasoR [39] was used in order to obtain the stem probability of the lincRNAs. Similarly, the dinucleotide shuffled sequences were used as background control. lincRNA secondary structure was examined with the three methods: RNAfold analysis based on MFE, ParasoR calculation of stem probability and calculation of folding strength based on a sliding window using RNAfold.

### Determination of sequence properties of lincRNAs

Custom scripts were used to determine properties of lincRNAs such as GC content and length. The getorf suite of EMBOSS was used to obtain open reading frames of each lincRNAs. Nucleotide sequences between START and STOP codons were considered valid ORFs. The longest ORF for each transcript from at least 25 aminoacids was selected.

The overlap of lincRNAs with transposable elements was analyzed using RepeatMasker [40] against species-specific repeat libraries. The distance between lincRNAs and their closest gene was obtained using BEDtools [41]. Also, lincRNAs were searched against the RFAM database using known covariance models of noncoding RNAs using the cmscan utility from Infernal [42]. Gene Ontology (GO) terms of all protein-coding genes were queried from Ensembl Metazoa or alternatively obtained using Blast2GO [43]. Enrichment of Gene Ontology terms was tested using Fisher’s test function under the topGO [44] package in R. Enrichment of GO terms of the closest genes for all lincRNAs in comparison to all protein coding genes of the species was analyzed to test whether lincRNAs tend to be located close to genes with certain functions. All statistical calculations and visualizations were obtained using R version 3.3.0 [45].

## Results and discussion

### LincRNAs show higher levels of GC content than intronic regions and generally do not cluster in certain regions of insect genomes

A total of 14,161 lincRNAs from seven different species were analysed (see Table 1). lincRNAs were classified into monoexonic and multiexonic for further study of their properties. lincRNA sequence length was variable in the different species ranging from a median of 544 nucleotides in *A. mellifera* to a median of 1006 nucleotides in *A.cerana* (see Additional file 1: Figure S1). lincRNAs were significantly shorter than coding sequence (Wilcoxon signed-rank test p-value <10^-06^ for all cases).

**Table 1:** LincRNAs used in this study

Species	Total lincRNAs	Monoexonic LincRNAs	Multiexonic lincRNAs	Reference
*T. castaneum*	1559	1327	232	Here assembled
*D. melanogaster*	2602	1807	795	[34, 63]
*A. gambiae*	2066	330	1735	[17]
*A. mellifera*	1529	310	1199	[24]
*A. cerana*	2459	379	2080	[24]
*N. vitripennis*	2176	431	1713	Here assembled
*D. pseudoobscura*	1770	655	1115	[25]

**Fig. 1:**
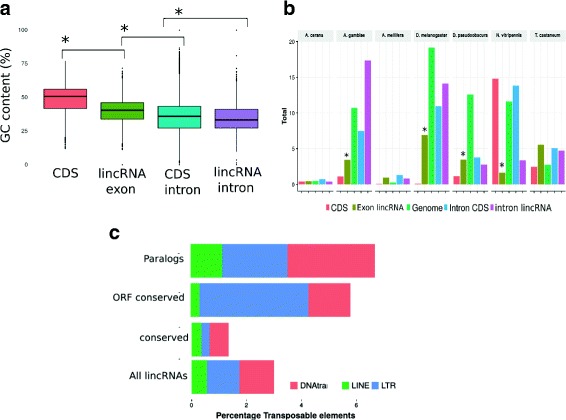
GC content and TE content of lincRNAs. **a** GC content of lincRNAs, lincRNA introns and coding sequences. LincRNAs have an intermediate GC content: higher than introns but lower than coding sequences. **b** Percentage of repeats of lincRNAs. LincRNAs have also an intermediate level of repeats. More repeats than coding sequences but less than introns. **c** Conserved lincRNAs have less TE. In contrast lincRNAs with signals of conservation in their ORF or paralogs have more TEs

lincRNA exons tend to show an intermediate GC content, i.e, lower GC content than protein coding exons (Wilcoxon test *p*-value<10^-8^) but higher than lincRNA introns (Wilcoxon test *p*-value<10^-8^ Fig. 1 a) as observed by [46]. In addition, multiexonic lincRNAs had overall a higher GC content than monoexonic lincRNAs (Wilcoxon test *p*-value<10^-6^, Additional file 1: Figure S2) although this pattern was not evident in all species. Furthermore, the number of exons per multiexonic lincRNA remains lower than in the case of coding transcripts as reported in several studies showing that length and number of exons tend to be lower in lncRNAs compared to protein coding genes [16, 20, 47].

High GC content is considered a hallmark of protein coding exons [48]. Sequence composition affects folding stability of RNAs. GC rich sequences tend to fold into more stable secondary structures. Indeed we observed for all species a highly significant correlation between GC content and thermodynamic stability and folding strength (Spearman’s rho =0.239, *p*-value <10^-16^ for all lincRNAs considering folding strength) as observed in [49]. Since lincRNAs tend to have higher GC content than introns and unconstrained intergenic sequences [46] (see Fig. 1 a) the higher GC content might be related to a higher tendency to form stable structures. Thus selection for GC content might be related to an increase in functionality, i.e, transcriptional efficiency and/or structural stability. We evaluated the correlation of GC content, folding strength and thermodynamic stability with expression of lincRNAs and protein coding genes in *T.castaneum* (See Additional file 1: Table S1–S2) where gene expression data from different tissues was available. For protein coding transcripts average gene expression and expression breadth are significantly although weakly correlated with observed folding strength and with Z-scores of folding strength which indicates that strongly folded transcripts tend to be expressed at a higher level. In contrast no remarkable difference was observed for lincRNAs (see Additional file 1: Table S2). lincRNAs have much weaker expression levels than protein coding genes, nonetheless this difference might be expected for some highly expressed lincRNAs.

In order to evaluate whether lincRNAs constitute indepependent transcriptional units from surrounding genes we tested whether transcription of lincRNAs was dependent on the surrounding genes. We observed that the Pearson’s correlation in expression of gene-lincRNA pairs is higher when gene-lincRNA pairs are at a closer distance (see Additional file 1: Figure S3A). We performed this test in *T. castaneum* where expression data for different tissues was available.

Genes with similar expression patterns tend to cluster in the genome [50]. To test if certain regions of the genome might be enriched in lincRNAs we looked at the chromosomal distribution of lincRNA in the genomes of *A.mellifera, T.castaneum, A.gambiae and D. melanogaster*. The purpose of this analysis was to detect regions in the genome with a higher density of lincRNAs. The genomes of the above mentioned species are resolved at the chromosome level, i.e, many of scaffolds are grouped into a reasonable number of linkage groups. Thus, distribution of lincRNAs can be more accurately analysed and visualized. Nevertheless a clear enrichment pattern could not be detected. lincRNAs are distributed throughout the chromosomes of the analysed species in a rather homogenous fashion; it does not appear that any chromosome or chromosomal region is significantly enriched or depleted in lincRNAs (Additional file 1: Figure S3B).

### Transposable elements (TE) could be a source of functional elements for lincRNAs

Transposable element content of lincRNA exons and lincRNA introns was analysed and compared to the transposable elements observed in coding sequences. A depletion of transposable elements in exons of lincRNAs compared with introns was found. This is indicative of purifying selection in exons, consistent with previous work (Wilcoxon test *p*-value <10^-6^ for *A.gambiae, D.melanogaster* and *N.vitripennis* (Fig. 1 b) [11]). The contribution of TE to lincRNAs was variable in the different species. Most notably, the hymenopterans *A. mellifera* and *A.cerana* contained almost no TE sequences as *Apis* genomes are also highly depleted in transposable elements [51]. The classes of TEs were also variable between the different species; for examples, *T.castaneum* showed an enrichment of DNA transposons compared to all other TE types whereas *D.melanogaster* had a comparatively higher content of long terminal repeats (LTR) which reflects the different transposable element contents of the genomes (Fig. 1 b and Additional file 1: Figure S4). Furthermore, *N.vitripennis* had a relatively low TE content in lincRNA exons indicating that the hymenopterans (*A.mellifera*, *A.cerana* and *N.vitripennis*) contained much fewer transposable elements in their lincRNAs compared to the dipterans (*A.gambiae*, *D.melanogaster*, *D.pseudoobscura*) or coleopterans (*T.castaneum*).

Even though repetitive sequences are generally selected against when TEs integrate into lincRNAs they can provide new domains and functions to the lincRNAs such as binding sites for proteins or structural elements [10]. The contribution of TEs to the evolution and properties of lincRNAs appears more important in vertebrate species with high transposable element content and more complex transcriptomes [10, 52]. The varying content of TEs in insect lincRNAs may indicate that similar processes may have also contributed to their emergence in at least some insect taxa. A recent study examined the factors contributing to the evolution of lincRNA in plants and their general properties [53]. In plants, the contribution of TEs is more modest; in contrast, duplication events (both local and whole genome duplication) appear to contribute to a larger extent to the evolution of lincRNAs. In insects, local duplication of lincRNAs could also be a major factor for the evolution of lincRNAs. Thus, repetitive sequences constitute, albeit to a lesser than in mammalian species, constitute a factor driving the evolution of lincRNA repertoires.

### Some lincRNAs show signs of structural stability

**Fig. 2:**
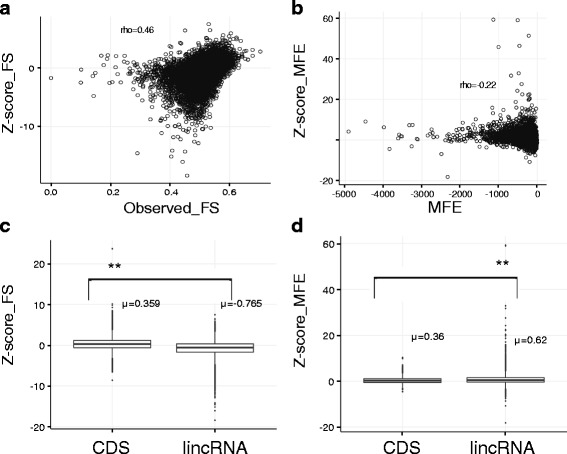
Secondary structure analysis of lincRNAs. **a** Distribution of Z-scores with folding strength (FS). Both are highly correlated (rho=0.46, *p*-value 2.2e16) which indicates that strongly folded sequences also tend to be more stable than their shuffled controls. **b** Distribution of Z-scores obtained from MFE of lincRNAs compared to shuffled sequences. A negative correlation (rho=-0.22, *p*-value 2.2e16) indicates than thermodynamically stable sequences (i.e longer because MFE scales with length) have higher Z-scores although several short sequences outliers with very strong Z-scores are present. **c** Comparison of Z-scores for FS obtained between lincRNAs and 10000 CDS from the seven species. CDS shows significantly bigger FS than lincRNAs. **d** Comparison of Z-scores for MFE calculations obtained between lincRNAs and 1O000 CDS from the seven species. Z-scores are significantly higher for lincRNAs

Comparison of mininum free energy (MFE) of the native lincRNA with mononucleotide and dinucleotide shuffled controls indicated that lincRNAs tend to be more stable (Wilcoxon test *p*-value <10 ^-8^) than the shuffled controls (Fig. 2 a). Another approach for determining secondary structure was carried out using ParasoR [39], a recent method that allows the determination of structural constraints on single sequences. Similarly as for RNAfold, the ratio of stem probability between the native lincRNA and the dinucleotide control was calculated. The ratio obtained with ParasoR and the ratio obtained with RNAfold were highly correlated (Spearman’s rho =0.68–0.80 for all species).

Our finding that lincRNAs have a lower MFE than the shuffled controls is an indication of a thermodynamically stable structure (Fig. 2 b–d). However, specially for longer sequences, the probability of the MFE is very low since the ensemble of secondary structures scales dramatically with the length of the sequence. The folding strength gives information on how frequently each nucleotide site is paired in the ensemble of secondary structures as described by [38]. We did not find lincRNAs to have larger folding strength than shuffled nucleotide sequences using this approach, in contrast to previous results found in human lincRNAs. (Figure 2 a–c) (Yang et al., 2015). However, some lincRNAs show very high folding strength. lincRNAs with strong folding strength and being thermodynamically very stable might have functions related to the secondary structure (Additional file 1: Table S3). We calculated the Z-scores of folding strength and thermodynamic stability for all lincRNAs using 100 nucleotide shuffled controls. The correlation between both measures was moderate (Spearman’s rho 0.22, *p*-value<2.2e.16) which indicates that different results might be obtained when considering only the MFE and the whole structural ensemble. We compared the Z-scores obtained with 10000 coding sequences obtained from the seven species and observed that lincRNAs have a higher Z-score than CDS when only considering the MFE but a lower Z-score when analysing the folding strength. This might be an effect of selection for maintaining codon triplets in coding sequences but it might also indicate that different selective constraints operate in both lincRNAs and CDS for the maintenance of secondary structure [38] (Fig. 2).

To obtain a confident set of structured lincRNAs (stlincRNAs) we selected the lincRNAs with the largest folding strength. We selected several quantiles, i.e, 90th, 95th, 97th, 99th of lincRNAs and analysed their properties. The main focus is to understand if highly structured lincRNAs present differences in comparison to other lincRNAs. stlincRNAs appear to be enriched in transposable elements (t-test *p*-value <0.05). DNA transposons, LINES and LTRs show signals of enrichment (Additional file 1: Figure S5A). There is an enrichment of LTR in structured lincRNAs which indicates that LTR are an important element conferring stable secondary structures to lincRNAs (2.07 percent in structured lincRNAs vs 1.17 percent in the total lincRNAs, (t.test *p*-value <0.05)). In contrast, we did not find an enrichment of low complexity regions or simple repeats in stlincRNAs (Additional file 1: Figure S5B). Furthermore, an enrichment of RFAM domain hits in the set of stlincRNAs was detected (t-test *p*-value <0.05). This validates the existence of stable and potentially functional RNA structures in at least some lincRNAs.

Structured lincRNAs are interesting candidates for further studies. Evidence of higher thermodynamic stability and/or folding strength and stronger folding than shuffled controls coupled with a confident match to a known covariance model (CM) from RFAM indicates that the secondary structure might be important in that particular lincRNA (see Additional file 1: Table S4). For example, lincRNA TCONS_00057427 from *T.castaneum* seems to possess a very stable secondary structure and a confident hit to a RFAM secondary structure model (RF01787).

### Very low conservation of lincRNA sequences in insect species

Sequence conservation of lincRNAs was assessed initially by BLASTN to detect regions on closely related species genomes where a confident match with the lincRNA was present. Homologous sequences to lincRNAs were defined as patches of BLAST matches of longer than 100nt and an e-value <10 ^-5^. Sequence conservation was observed only in close-related species. Above 50Mya of evolutionary distance, sequence conservation is in most cases difficult to detect. Furthermore, homology of a lincRNA to another genome does not indicate that the homologous locus is transcribed [4]. Thus, we focused on homology detection within lincRNA sequences. Homologous hits were mostly detected between *A. mellifera* and *A. cerana* lincRNAs and between the two *Drosophila* species. The divergence time of the two *Apis* species is estimated to be 18.5 million years (Mya) as reported on TimeTree [54] whereas the divergence between the two *Drosophila* species is around 33 Mya. The other species diverged well over 100 Mya [54] thus considering the generally observed low-levels of sequence conservation of lincRNA it is not surprising that the levels of conservation are small [4].

**Fig. 3:**
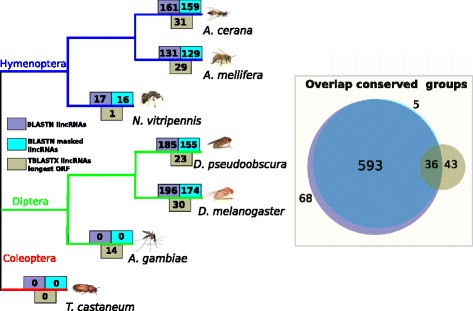
Conservation analysis of lincRNAs in the seven studied species. Comparison of lincRNAs exons was performed using BLAST with the native lincRNAs, with the sequences of the lincRNAS with masked repeats and with the longest ORF of all the lincRNAs. A low sequence conservation was observed for lincRNAs in insects. Five hundred ninety three lincRNAs were observed conserved in their nucleotide sequence both with masking and without masking repeats. Furthermore 43 lincRNAs showed signals of conservation in their ORFs and 68 showed indication of conservation only without masking repeats

To analyze in more detail the conservation of lincRNAs between all the different species and obtain clusters of conserved sequences we performed all-vs-all BLASTN and MCL clustering of the corresponding BLAST results (see Fig. 3). A total of 690 lincRNAs were detected as conserved between at least 2 of the 7 species (see Fig. 3). Most of the conserved lincRNAs were between the two Apis species (292) and the two Drosophila species (381). This is an indication of the fast sequence evolution of lincRNAs. Beyond 50MyA there is mostly no conservation on the majority of lincRNAs. Notably no conserved lincRNAs based on BLASTN were detected in *Tribolium castaneum* and *Anopheles gambiae*. To test whether the conservation measures were driven by transposable elements and low-complexity regions we performed the same conservation analysis after masking repeats and low-complexity sequences. A total of 633 clusters were obtained using this approach. The majority of lincRNAs with conservation related to transposable elements, simple repeats or low complexity regions were lincRNAs from *D.melanogaster* or *D.pseudoobscura* (91.22%).

In some cases, conserved lincRNAs could be remnants of protein coding genes as previously described [4] and also unannotated UTRs or regulatory elements. To test whether lincRNA evolution was influenced by the proximity to a protein coding gene we analyze the proximity to a protein coding gene in the conserved lincRNAs. Conserved lincRNAs did not appear to be closer to the nearest gene. Thus it is likely that lincRNAs form independent transcription units from surrounding genes. We also evaluated whether conserved lincRNAs tend to show stronger folding. It does not appear that conserved lincRNAs have a stronger folding (see Additional file 1: Figure S6A).

A similar BLASTN approach was used with the aim of detecting paralog lincRNAs. Paralog lincRNAs are here defined as lincRNAs with sequence similarity within the same species (e-value < 10 ^-5^) (see Table 2). Using this approach we found paralog stretches in the different species: 768 *T. castaneum* lincRNA (49.3%); 356 lincRNA in *A.cerana* (14.4%); 83 in *A. mellifera* (5.4%), 281 in *N. vitripennis* (12.9%); 422 in *A. gambiae* (20%), and 506 in *D.pseudoobscura* (28.6%).

**Table 2:** LincRNAs with signals of conservation in other species (labelled as conserved); with paralog streches (paralogs) and containing overlaps with transposable elements in their spliced exonic sequences (transposable element derived)

Species	Total lincRNAs	Conserved (after masking repeats)	Paralogs	TE related	Structured
*T. castaneum*	1559	0	768	827	37
*D. melanogaster*	2602	174	869	443	79
*A. gambiae*	2066	0	422	237	180
*A. mellifera*	1529	129	83	1	80
*A. cerana*	2459	159	356	0	56
*N. vitripennis*	2176	16	259	109	205
*D. pseudoobscura*	1770	155	506	246	72

A significant fraction of the similarity detected between lincRNAs within species is expected to be due to the presence of TEs repeated in multiple copies in the genomes studied. TEs could have a role in the expansion of the repertoire of lincRNAs [10] as reported in vertebrate species [11]. Indeed we observed a significant overlap of TEs in the set of paralogous lincRNAs compared to the rest (Additional file 1: Figure S7). Paralog lincRNAs tend to have more exons and tend to be longer (except *T. castaneum*). Paralog lincRNAs appear not to be located at a significantly different distance from a protein-coding gene on average in comparison with all lincRNAs.

If transposable elements were an important element conferring secondary structure to lincRNAs we would expect a higher structural stability in the lincRNAs of *Anopheles, Nasonia* and *Tribolium* compared to the two *Apis* species. However we do not see that lincRNAs from *A. mellifera* and *A. cerana* are less stable than the lincRNAs of the other species. However, *A.gambiae* lincRNAs appear more strongly folded than lincRNAs from the other species (Wilcoxon *p*-value<2.6* 10^16^) (see Additional file 1: Figure S6B). Stronger thermodynamic stability of some lincRNAs might also be explained by elements conferred by short non-coding RNAs such as microRNAs or snRNAs [55].

Some lincRNAs might act on cis by regulating close proximity genes. Thus, knowledge of the functions and biological processes of the genes which are located in close proximity to lincRNAs is important to find potential functions of lincRNAs. The enrichment of GO terms from genes closest to lincRNAs compared to all genes for each species was evaluated (Additional file 2: Table S5). No remarkable enrichment was found. This was rather expected as lincRNAs are rather equally distributed throughout the genome. However, more information can be obtained when analysing subsets of potentially functional lincRNAs such as structured lincRNAs or conserved lincRNAs. Thus, GO enrichment of the closest genes to each lincRNAs classified as potentially structured was analysed (Additional file 3: Table S6). Some terms appear enriched in the structured lincRNAs such as ion binding in *N.vitripennis* (*p*-value 0.00031) or nucleotide binding in *D.pseudoobscura* (*p*-value 1.5e-29).

### Some lincRNAs might be precursors for the emergence of *de novo* protein coding genes.

Some characteristics might favour lincRNAs to bind to the ribosome and be ultimately translated. Such characteristics include mRNA-like features including capping and 5’UTR length [56]. lincRNAs could rise to short functional ORFs [57, 58] or to protoORFs and ultimately to a new protein coding gene [59]. lincRNA transcription and in some cases translation provide a substrate for evolution to produce genetic novelty in the form of emergence of *de novo* genes. Some properties of lincRNAs might favour some of them to act as precursors for *de novo* genes (see [60]). *De novo* genes tend to be short and contain generally less exons than protein coding genes. *De novo* emerged genes tend to be highly enriched in disordered regions [61]. High GC content of nucleotide sequences is also known to be correlated with intrinsic disorder of proteins since high GC increases frequency of Gly, Ala, Arg, and Pro aminoacids that are more represented in disordered regions of proteins [62]. Thus, GC-rich lincRNA and lincRNA with repetitive sequences might be an important place to look when mining for the presence of *de novo* emerged genes from intergenic sequences [19].

We analysed lincRNA open-reading frames (ORFs) to test for signatures of conservation at the protein level and to evaluate the properties of lincRNA ORFs. We applied all-vs-all tBLASTX of the ORFs to detect signatures of selection on the lincRNAs related to amino acid conservation. MCL clustering of the BLAST results was performed to detect lincRNAs with conservation at the amino acid level. A smaller number of lincRNAs showed signals of conservation when analysing the longest ORF than by analysing nucleotide sequences (Fig. 3). Surprisingly, 14 lincRNAs were detected to be conserved in *A. gambiae*; whereas no lincRNA had been detected to be conserved when analysing the RNA sequence. After examining in more detail the lincRNAs in *A.gambiae* a strong indication of protein coding potential was found. A total of 241 lincRNAs were suspected to be protein-coding genes or fragments of pseudogenes since they contained a significant BLASTX hit (e-value <10^-05^) to the Uniref90 protein database. A total of 733 lincRNAs from *D.melanogaster* also had a significant hit against protein databases. Some studies filter out lincRNAs with signals of similarity to known proteins or protein domains whereas others are less strict [17, 63]. A strict filtering was applied to remove lincRNAs with similarity to protein coding genes for *A.mellifera*, *A.cerana* and *D.pseudoobscura* [24, 25]. We applied a similar strategy to obtain a confident set of lincRNAs from *T.castaneum* and *N. vitripennis*. In contrast, lincRNAs from *D. melanogaster* and *A.gambiae* were not so stringently filtered [17, 63]. A clear consensus on the definition of a lincRNA would avoid mistaken annotations and comparable lincRNA datasets. Ideally comparable filtering steps should be applied on assembled transcripts between different studies to remove pseudogenes, UTRs and fragments of coding exons. A consistent strategy should be defined to differentiate lincRNAs, pseudogenes, unannotated gene fragments, transcriptional noise and potentially emerging *de novo* genes obtained from RNA-seq data.

## Conclusions

We presented the first study analysing properties of lincRNAs in multiple insect species. The approach presented here used RNA-seq data to assemble lincRNAs from *T. castaneum* (Additional file 4) and *N. vitripennis* (Additional file 5) and used previous lincRNAs assembled in previous studies from *A. gambiae*, *A. mellifera*, *A. cerana*, *D. melanogaster* and *D. pseudobscura*. The numbers of lincRNAs obtained in each study were variable, similarly as the tissues and RNA-seq read depths used for the assembly. However, evidence of transcription gives confidence on the validity of the lincRNA annotations. We defined lincRNAs based on transcriptional evidence from RNA-seq studies. An alternative strategy in order to find orthologs of well-known lncRNAs in close relative species was recently explored in the drosophila clade [5] using microsynteny, sequence and secondary structure conservation. In the proposed species sequence divergence, genome rearrangements and much weaker stuctural conservation signals impede such analyses.

We showed that lincRNA sequence conservation is very low and is almost undetectable beyond 50Mya of evolution for most lincRNAs. However, lincRNA exons, in contrast to their introns, present properties such as high GC content or lower transposable element content which are more similar to protein coding genes. Furthermore, some lincRNAs might have structural constraints. These observations indicate that the function of several lincRNAs in insects might be related to their secondary structure.

New and innovative approaches to understand the role of lincRNAs in different biological processes are required. The presence of conserved motifs on RNA sequences points to the necessity of strategies of detecting them and better establishing the functional relationship between sequence, structure and function in lincRNAs. Recent technological innovations such as Parallel analysis of RNA structure [64] which allow determination of secondary structures from nucleotide sequences will allow improvement of bioinformatics algorithms for prediction and comparison of RNA structures that will definitively help characterise those lincRNA with structural contraints.

## Additional files

Additional file 1 Additional Figures and Tables. Additional Figures S1-S7 and Table S1-S4. (PDF 1997 kb)

Additional file 2 Table S5. (ODS 28 kb)

Additional file 3 Table S6. (ODS 26 kb)

Additional file 4 Annotations of *T.castaneum* lincRNAs. (GFF 190 kb)

Additional file 5 Annotations of *N.vitripennis* lincRNAs. (GFF 179 kb)

## Abbreviations

A.cerana, A.ce: Apis cerana
A.gambiae, A.gam: Anopheles gambiae
A.mellifera, A.me: Apis mellifera
BLAST: Basic local alignment search tool
CM: Covariance model
D.melanogaster, D.me: Drosophila melanogaster
D.pseudobscura, D.pse: Drosophila pseudobscura
FS: Folding strength
GO: Gene ontology
LncRNA: Long noncoding RNA
lincRNA: Long intergenic noncoding RNA
LTR: Long terminal repeats
MFE: Minimum free energy
Mya: Million years ago
N.vitripennis, N.vit: Nasonia vitripennis
ORF: Open reading frame
SRA: Sequence read archive
TSS: Transcription start sites
TE: Transposable element
T.castaneum,T.ca: Tribolium castaneum
