# Supplementary material for: Comparative analysis of lincRNA in insect species
Source: BMC Evol Biol. 2017 Jul 3;17:155. doi: 10.1186/s12862-017-0985-0 (PMC5494802; doi:10.1186/s12862-017-0985-0)
Supplement: Supplementary file 1 — Additional Figures and Tables. Additional Figures S1-S7 and Table S1-S4. (PDF 1997 kb) [file 12862_2017_985_MOESM1_ESM.pdf]

Supplementary Figures

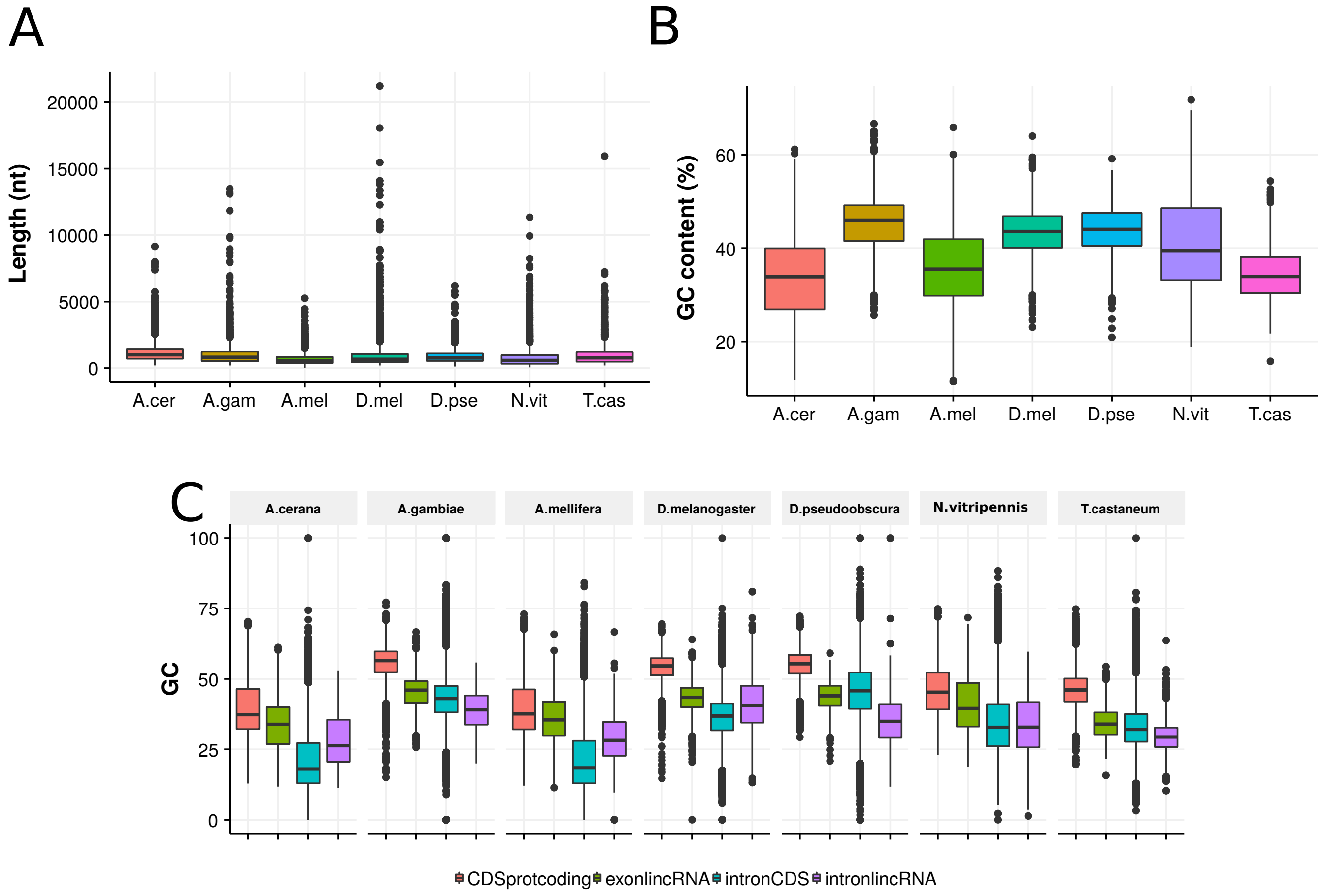

Figure S1: A) Length of the different lincRNAs for the seven species studied. *D.melanogaster* contain some very long lincRNAs B) GC content of lincRNA exons for the seven insect species. C) GC content of CDS, lincRNA exons, lincRNA introns and introns protein coding exons. lincRNAs have an intermediate level of GC content higher than both protein coding and lincRNA introns but lower than protein coding exons.

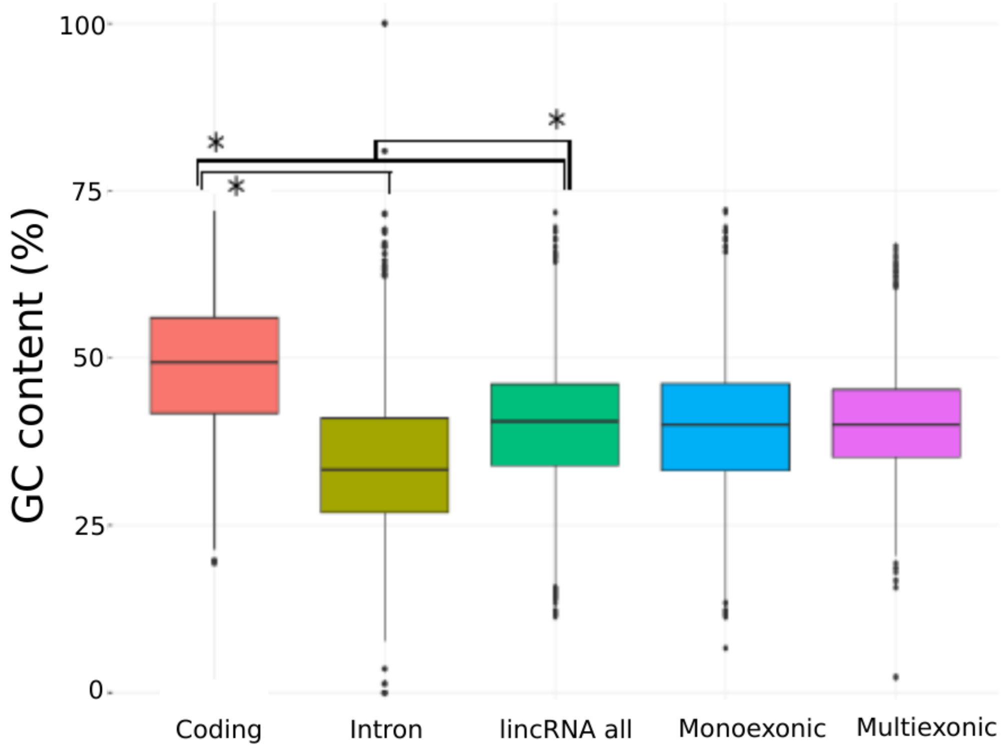

Figure S2: GC content of lincRNAs in coding sequences, lincRNA introns, monoexonic lincRNAs and multiexonic lincRNAs. lincRNA exons have higher GC content than introns but less than coding sequences(CDS).

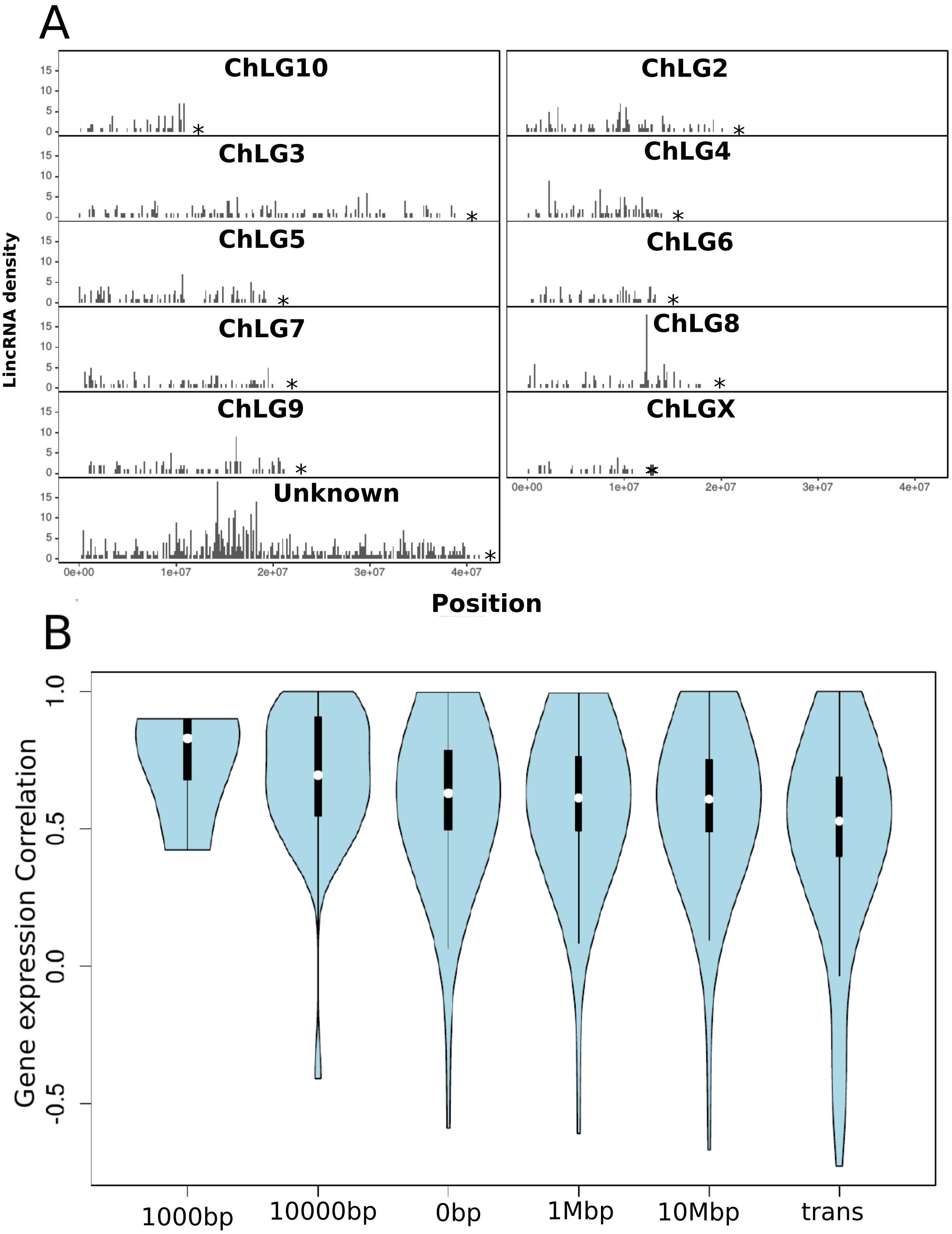

Figure S3: A) Distribution of lincRNAs in *T.castaneum* genome. The end of the chromosome is represented as astherisk (*). LincRNAs do not appear to cluster in the genome of insect species. B) LincRNAs show a greater correlation on expression to genes that are in their vicinity. The effect decreases as the distance increases. Genes that show similar expression patterns tend to cluster in the genome and we observe that lincRNAs also follow that pattern.

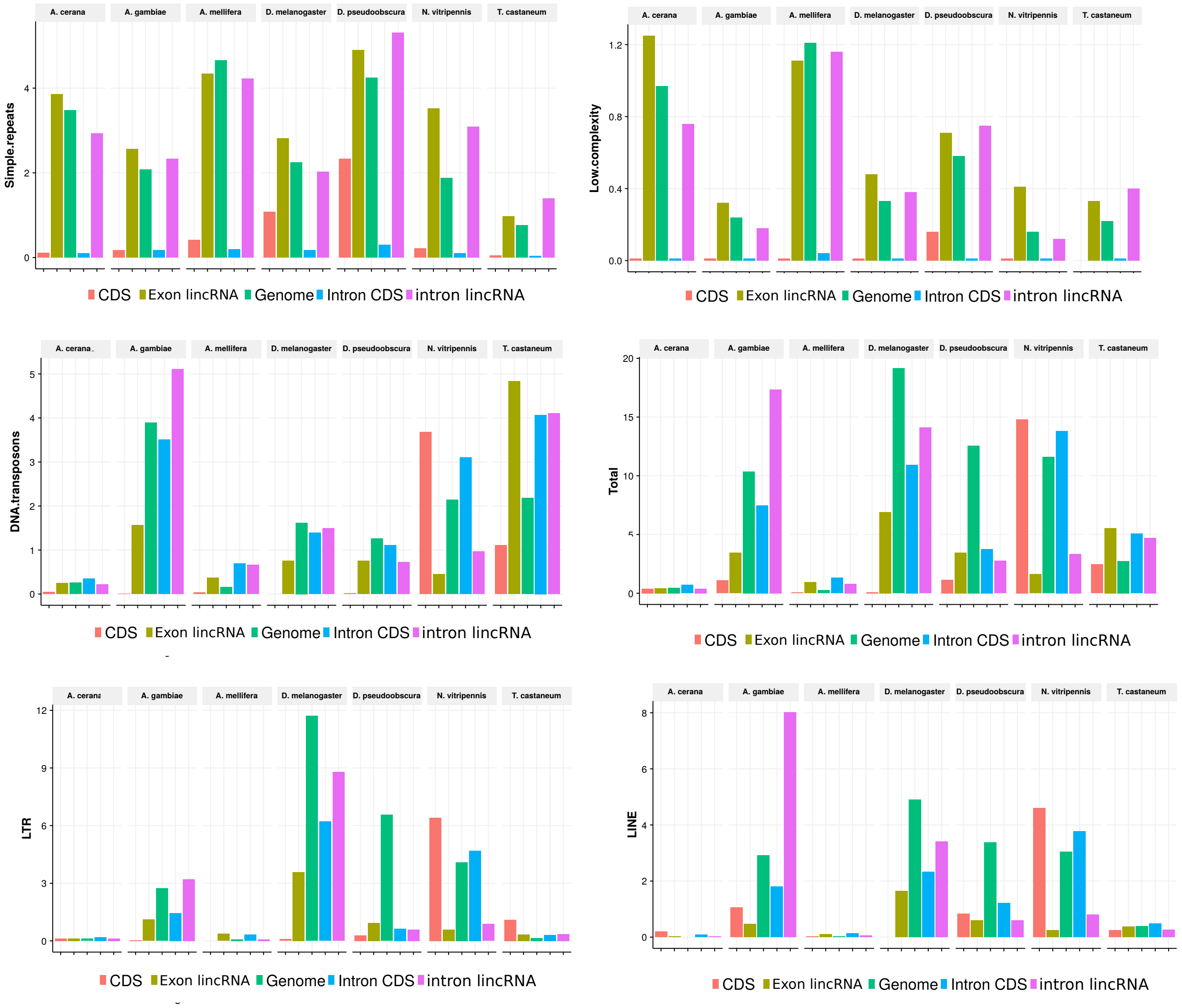

Figure S4: Transposable element content of lincRNAs exons, lincRNA introns, protein coding exons, protein coding introns and genome. LincRNA exons tend to be depleted in comparison to introns. Coding sequences tend to be strongly depleted in insertions of transposable elements. *N. vitripennis* represents an exception since an important number of coding sequences are derived from repetitive elements. The distribution of different types of transposable elements i (LINE, LTR, DNA transposons and total of known repetitive sequences) and low complexity and simple repeats for the different regions are shown

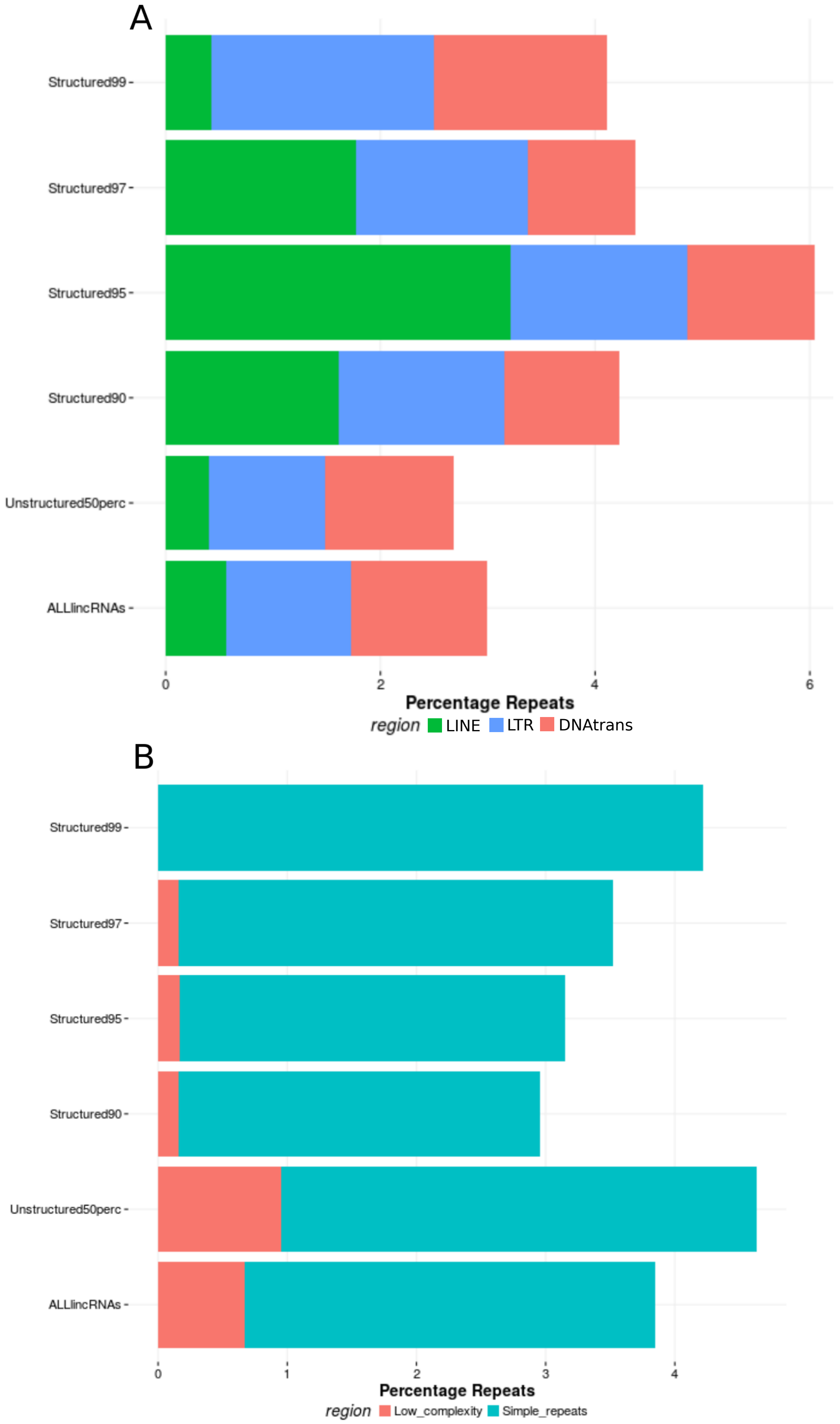

Figure S5: Transposable elements (A), low complexity regions and simple repeats (B) of all lincRNAs compared to structured lincRNAs obtained by RNAfold folding using a sliding window approach to obtain the folding strength of the lincRNAs. Structured lincRNAs are enriched in transposable elements; specially specially LINEs and LTR elements. Structured90, Structured95, structured97 and structured99 represent the lincRNAs that have a higher folding strength than the other 90,95,97 and 99 percent of the rest lincRNAs. In contrast unstructured50 correspond to lincRNAs that are less folded than the 50% of the rest lincRNAs.

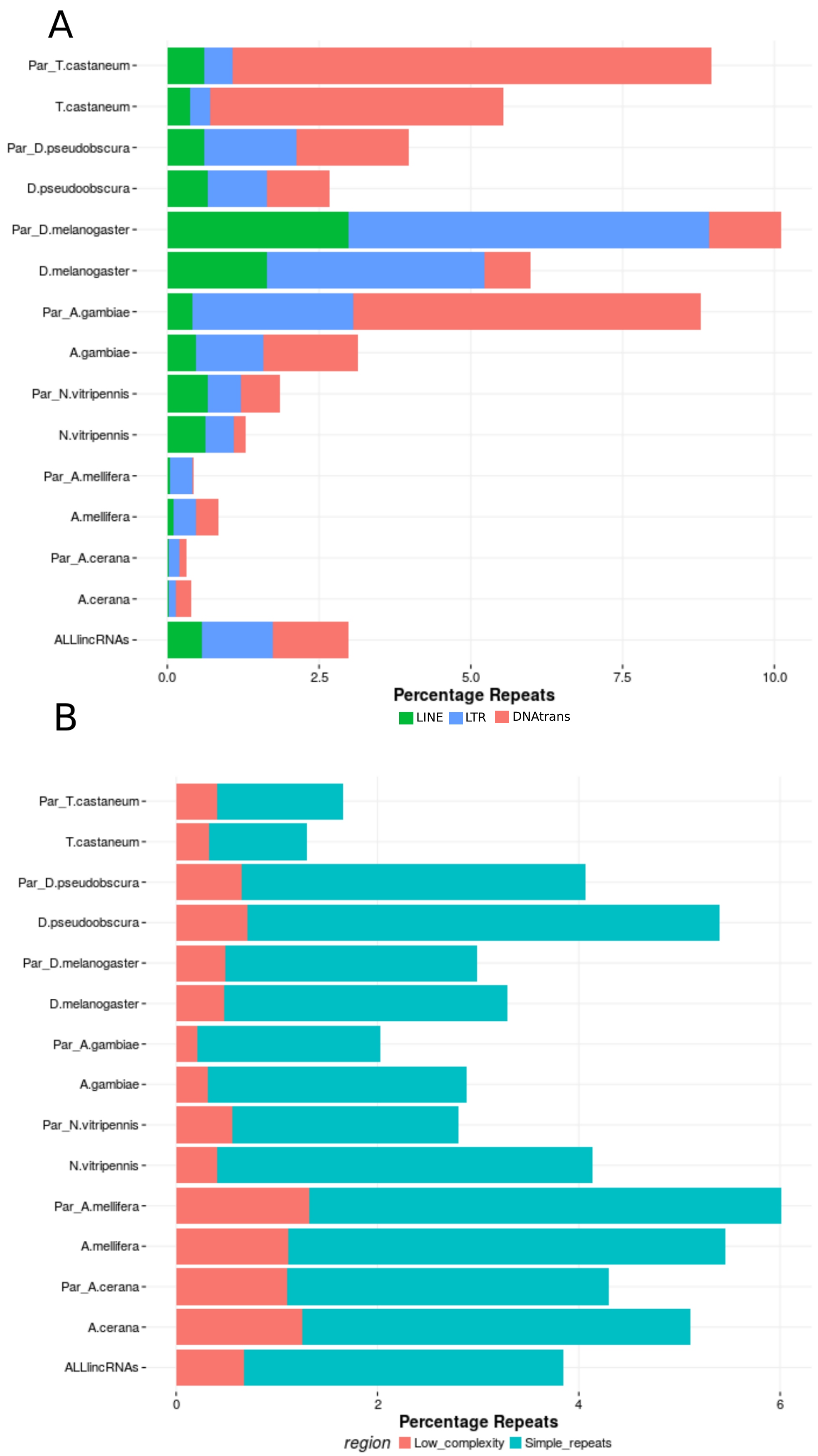

Figure S6: A)Percentage of repeats for the seven species exonic lincRNAs and for the paralog lincRNAs obtained after MCL clustering of the allvsall BLASTN results of the lincRNAs. Paralog lincRNAs are generally enriched in transposable elements. Similarity in-between species lincRNAs is caused by transposable elements. B) Percentage of low complexity regions in lincRNAs exons for each species and for the paralog lincRNAs of the same species based on the MCL clustering of BLASTN results.

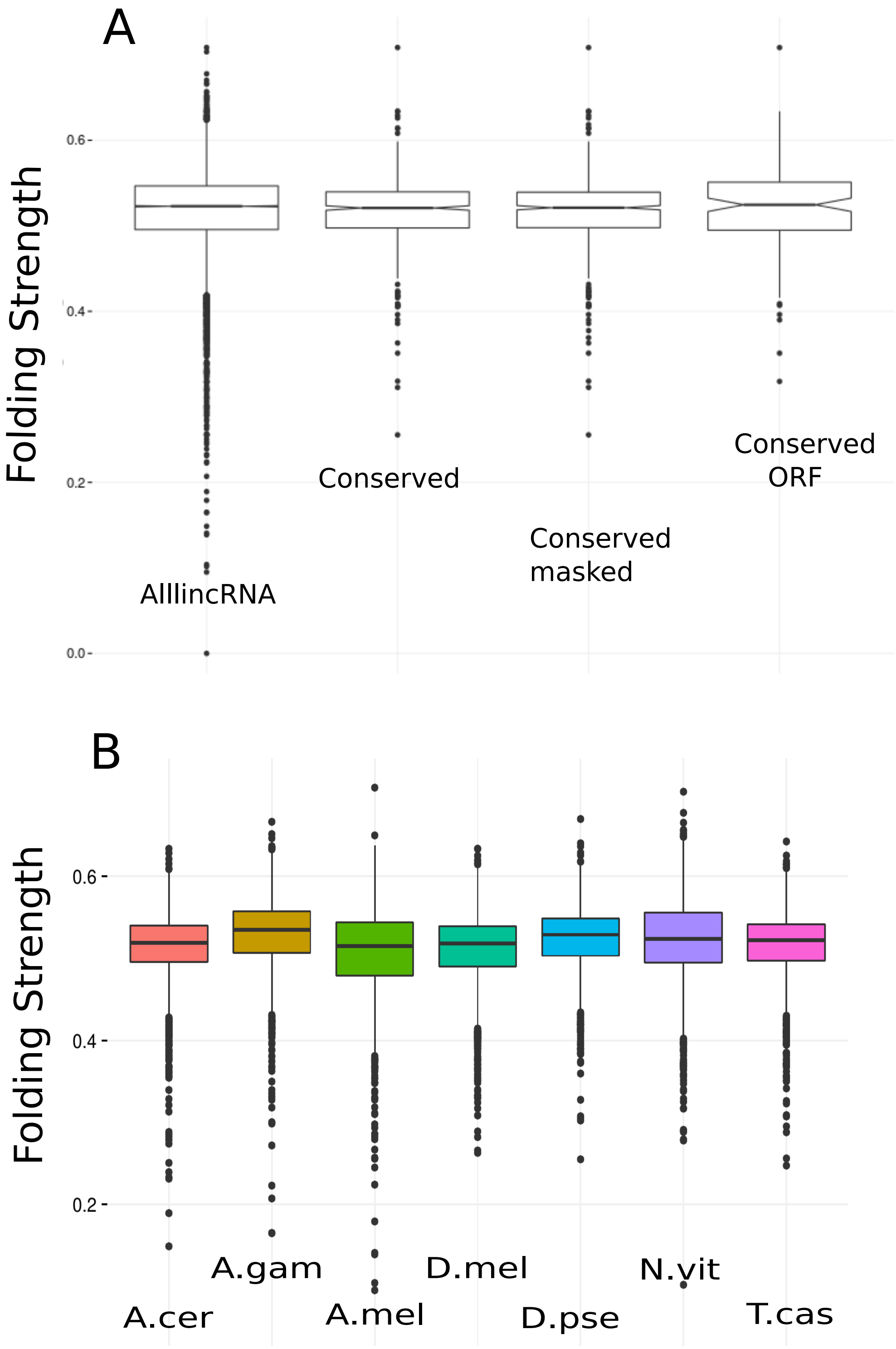

Figure S7: A) Fold strength of the different lincRNAs. Conserved lincRNAs do not appear to have a greater tendency to be folded than the rest of lincRNAs. B) Fold strength of the different lincRNAs for the seven species. Differences between the folding strengths of the lincRNAs of the different species occur in parallel as differences in GC content i.e *A. gambiae* is a GC-rich genome and the hymenopterans *A.mellifera*, *A.cerana* and *N.vitripennis are AT-rich genomes*

Supplementary Tables

Table S1: Correlation expression protein coding genes in *T.castaneum* with RNA structural parameters. Expression average corresponds to average RPKM of expression across 8 tissues. Expression breadth is the fraction of tissues with detected RPKM>0. Observed folding strength (FS) is the percentage of paired nucleotides for the sequence used as a proxy for the identification of strongly folded sequences. Z-score FS is obtained after Z-transformation of observed FS compared to 100 shuffled controls.

**CDS *T.castaneum***	Correlation measure	Expression Average	Expression breadth
Observed FS	Spearman's correlation	*0.065 (p-value 4.7e-12)	*0.047 (pvalue 5.28e-7)
	Partial correlation (control length)	*0.073 (p-value 7.20e-15)	0.050 (p-value 6.791e-08)
	Partial correlation (control GC content)	*0.024 (pvalue 0.0093)	0.01154 (p-value 0.220)
SD(FS)	Spearman's correlation	*0.45 (p-value <2.2e-16)	*0.39 (p-value <2.2e-16)
	Partial correlation (control length)	*-0.066 (p-value 1.12e-11	*0.063 (p-value 1.195382e-11)
	Partial correlation (control GC content)	*0.452673 (p-value 0)	*-0.389 (p-value 0)
Zscore(FS)	Spearman's correlation	-0.014 (p-value 0.15)	0.011 (pvalue 0.26)
	Partial correlation (control length)	*0.054 (p-value 7.09e-09)	*-0.024 (p-value 0.0090)
	Partial correlation (control GC content)	*0.0215 (p-value 0.022)	-0.0063 (p-value 0.4993)

Table S2: Correlation expression lincRNAs with RNA structural parameters. Expression average corresponds to average RPKM of expression across 8 tissues. Expression breadth is the fraction of tissues with detected RPKM>0. Observed folding strength (FS) is the percentage of paired nucleotides for the sequence uses as a proxy for the identification of strongly folded sequences. Zscore FS is obtained after Z-transformation of observed FS compared to 100 shuffled controls.

**lincRNA *T.castaneum***	Correlation measure	Expression Average	Expression breadth
Observed FS	Spearman's correlation	0.029 (p-value 0.23)	0.0186 (pvalue 0.461)
	Partial correlation (control length)	0.0177 (p-value 0.48)	0.013 (p-value 0.59)
	Partial correlation (control GC content)	0.028 (p-value 0.25)	0.017 (p-value 0.49)
SD(FS)	Spearman's correlation	*-0.11 (p-value 1.19e-05)	-0.075* (p-value 0.0027)
	Partial correlation (control length)	-0.018 (p-value 0.46)	-0.045 (p-value 0.075)
	Partial correlation (control GC content)	*-0.1-1 (p-value 1.05e-05)	-0.075 (p-value 0.0030)
Zscore (FS)	Spearman's correlation	-0.041 (p-value 0.1027)	0.044 (p-value 0.077)
	Partial correlation (control length)	*0.064 (p-value 0.010)	*-0.056 (p-value 0.0025)
	Partial correlation (control GC content)	0.041 (p-value 0.10)	0.046 (p-value 0.068)

Table S3: Selected lincRNAs corresponding to the 99.8 percentile of lincRNAs based on folding strength (FS). Asterisk (*) indicates conservation based on BLAST to other species lincRNAs. Minimum free energy (MFE) of the most stable structure for each lincRNA and for the dinucleotide shuffled sequences (MFEs). LincRNAs were selected based on folding strength i.e percentage of paired nucleotides in the structure ensemble; however it also occurs that some lincRNAs have smaller MFE than their shuffled controls.

Sequence Name	Observed_FS	Shuffled_FS	Lenght	GC%	MFE	MFEshuf
AGA_Merged.10515.1	0.633639435226737	0.612773352455892	378	42.06	-97.7	-100.2
AGA_Merged.11140.1	0.646707932401574	0.627275301769522	346	47.69	-119.2	-105.9
AGA_Merged.13866.1	0.666725895363666	0.652755010727147	323	58.2	-131	-120.3
AGA_Merged.13867.1	0.635176585827888	0.636500512402316	499	60.92	-227.1	-198.3
AGA_Merged.14568.1	0.635966089803476	0.627546368693786	658	59.88	-276.5	-262.1
AGA_Merged.18611.1	0.637019993321646	0.602097271415453	242	58.26	-83.4	-92.7
AGA_Merged.19566.1	0.634660781605226	0.602040870651982	270	63.33	-117.1	-113.1
AGA_Merged.19869.1	0.651780439487051	0.635279149225431	484	63.02	-211.6	-205.5
AGA_Merged.4488.1	0.632624042624043	0.548271564501073	305	53.77	-81.6	-92.8
AME_HP497041.1	0.638083920764644	0.559415433963627	332	47.29	-103.4	-98.4
*AME_HP498920.1	0.708428553478058	0.539397923308814	303	22.11	-99.4	-83.4
AME_HP536139.1	0.650136361895944	0.624251445418692	287	44.25	-89.9	-81.9
CER_AC_lincRNA.3601.1	0.633839786259141	0.613720604365766	465	56.77	-167.8	-167.4
*DME_DLt00031	0.633823868439253	0.601931039974518	299	55.85	-115.4	-112.3
DME_DLt00196	0.633823868439253	0.594908844407172	299	55.85	-115.4	-107.3
DPS_TCONS_00019395	0.6701407068996	0.567272670237097	253	52.96	-80.3	-75.2
DPS_TCONS_00035919	0.640557096625058	0.569983818770226	206	40.78	-52.9	-41.7
DPS_TCONS_00050381	0.63689331943859	0.585807463272253	1491	46.68	-511.5	-474.5
NAS_TCONS_00014495	0.648838010702418	0.597747458128814	354	59.32	-125.6	-123.5
NAS_TCONS_00014589	0.634333776459761	0.586035126291032	254	57.48	-100.7	-104.3
NAS_TCONS_00014627	0.642031927303245	0.507319811970975	387	56.59	-142.9	-132.2
NAS_TCONS_00016721	0.639823717948718	0.602747252747252	208	57.69	-66.9	-72.1
NAS_TCONS_00017749	0.651664400038384	0.610413532568004	369	58.54	-140.2	-149.5
NAS_TCONS_00018277	0.633941932409899	0.567704154403319	359	48.47	-113.2	-104.5
NAS_TCONS_00018278	0.649023765350296	0.606330800718556	245	31.43	-65.1	-56.6
NAS_TCONS_00030233	0.645434209719924	0.617030759887903	259	57.53	-91.1	-88.9
NAS_TCONS_00032485	0.652116402116402	0.630710978835979	240	37.92	-55.6	-53.8
TCA_TCONS_00069138	0.64270138108665	0.621640808043075	353	54.39	-120.5	-118.2

Table S4: Some lincRNAs with highly significant similarity to known RFAM motifs.

Species	Sequence name	RFAM	E-value	Length
T. castaneum	TCONS_00002931	Introngpl	0.0056	507
T. castaneum	TCONS_00057427	drz agam1	9.7e-05	1129
T. castaneum	TCONS_00067824	Histone3	0.00092	1910
A. mellifera	HP569333	ZNFx1-AS1_3	0.0053	729
A. mellifera	HP568152	RUF21	0.0024	970
A. mellifera	HP518143	U2	1e-0.33	1443
A. cerana	Ac_lincRNA_12491.1	RUF21	3.6e-03	757
A. cerana	Ac_lincRNA_71311.1	TMV UPDK3	2.3e-03	370
A. cerana	Ac_lincRNA_82226.1	GlsR8 mir10	6.4e-03	686
N. vitripennis	TCONS_00033992	SCARNA15	8.9e-03	309
N. vitripennis	TCONS_00026099	Entero Ori	6.6e-03	298

V
